# Mr. Grose, on the Vaccine Inoculation

**Published:** 1800-04

**Authors:** J. H. Grose

**Affiliations:** Winslow


### *To the Editors of the Medical and Phyſical Journal.*

Gentlemen,

HAVING had the pleaſure, ſome time ſince, to peruſe Dr. Jenner's Obſervations on the Cow-pox, wherein he aſcribes the origin of it to a diſeaſe in the horſe; and having heard thoſe remarks diſputed, I have taken the liberty of communicating ſome additional information.

I have had many opportunities of converſing with reſpectable farmers, whoſe cows were affected with the diſeaſe, and they unanimouſly agree in aſcribing it to a complaint in the horſe's heel, which is called, from its ſingularity of making the hair erect, "a ſcratchy heel." Now, there are many diſorders incident to the heel, which do not come under this deſcription; being well fed, or want of exerciſe, will frequently excite ſwellings, which are by no means connected with a ſcratchy heel. The ſpurious Cow-pox does not ariſe from this cauſe; but is frequently produced when cows, full of milk, are taken to fairs, and their bags permitted to remain full for a length of time: it will alſo ariſe from ſore teats neglected; and by the friction of the milker's hand, a quantity of extravaſated blood is carried on the fingers to the reſt of the cows, and produces, by abſorption, a diſeaſe ſimilar, but not exactly correſponding, with genuine Cow-pox. The real diſeaſe may always be diſ-

tinguiſhed tinguished by the appearance of "*concave scaly eruptions*" on the top of the teat, which is never produced by a spurious sort.

With respect to Dr. Jenner's assertion, that a person may be "*repeatedly affected, both locally and generally, with the Cow-pox*," I am sufficiently satisfied of the contrary by a variety of well-attested cases. A farmer, at some distance from this place, had the Cow-pox a few years past; and though he has been in the habit of milking cows, when the disease has appeared in its most virulent state, he has never experienced so much as an inflamed hand; nor has he received any infection from the Small-pox, though recently inoculated.*

I could recount numerous cases, to corroborate this assertion; and I have found them unsusceptible of either. If the Small-pox is ever received after being inoculated with vaccine matter, it must unquestionably arise from the latter not being genuine; as, in every case I have witnessed, where the person had been infected with Cow-pox, though at a distant period, the insertion of variolous matter has been productive of no effect.

Some medical gentlemen have been inclined to suppose, and have, indeed, ventured to assert, that the appearance of pustules in Cow-pox, has been occasioned by the unknown introduction of variolous matter; whereas, it has been proved that eruptions, similar to the Small-pox, had appeared, though a new lancet had been used. A convincing proof of this I witnessed not long ago, where a farmer (for all sorts of people inoculate here) inserted the vaccine matter on the point of an awl, and three out of five had eruptions to the number of twenty; three or four on the face and hands, but chiefly confined to the arm. These pustules were filled with matter; and others were inoculated from the pustules, experiencing the disease in its mildest form, and without any eruption. I know it has been supposed, that matter taken from pustules would produce them; but this is a striking proof to the contrary. They certainly did not arise from Small-pox, (as has been represented) but from a deep insertion of the virus. Indeed, a more valuable discovery cannot be made for the public than this, as it may be the means, under Providence, if not of banishing, at least diminishing, the fatal influence of a disorder which has so long desolated mankind; and I am happy to add that the practice is daily extending. I have the honor to be,

GENTLEMEN,

Your humble servant,

J. H. GROSE.

*Winslow,*

*March* 15, 1800.

* The gentleman alluded to, recollects the Cow-pox being known in the county as a preservative from Small-pox thirty-six years ago.

